# “There is a Place”: impacts of managed alcohol programs for people experiencing severe alcohol dependence and homelessness

**DOI:** 10.1186/s12954-019-0332-4

**Published:** 2019-12-16

**Authors:** B. Pauly, M. Brown, J. Evans, E. Gray, R. Schiff, A. Ivsins, B. Krysowaty, K. Vallance, T. Stockwell

**Affiliations:** 1 Canadian Institute for Substance Use Research, University of Victoria, Technology Enterprise Facility Room 273, 2300 McKenzie Ave, Victoria, BC V8P 5C2 Canada; 2 School of Nursing, University of Victoria, HSD Building A402A, Victoria, BC V8P 5C2 Canada; 3 Department of Earth and Atmospheric Sciences, University of Alberta, 1-26 Earth Sciences Building, Edmonton, AB T6G 2E9 Canada; 4 School of Social Work, MacEwan University, 9-505 Robbins Building, Box 1796, 10700-104 Avenue, Edmonton, AB T5J 2P2 Canada; 5 Department of Health Sciences, Lakehead University, 955 Oliver Road, Thunder Bay, ON P7B 5E1 Canada

**Keywords:** Managed alcohol programs, Homelessness, Harm reduction, Safer drinking interventions, Situational analysis, Illicit alcohol, Risk environment, Enabling environments

## Abstract

**Background:**

The twin problems of severe alcohol dependence and homelessness are associated with precarious living and multiple acute, social and chronic harms. While much attention has been focused on harm reduction services for illicit drug use, there has been less attention to harm reduction for this group. Managed alcohol programs (MAPs) are harm reduction interventions that aim to reduce the harms of severe alcohol use, poverty and homelessness. MAPs typically provide accommodation, health and social supports alongside regularly administered sources of beverage alcohol to stabilize drinking patterns and replace use of non-beverage alcohol (NBA).

**Methods:**

We examined impacts of MAPs in reducing harms and risks associated with substance use and homelessness. Using case study methodology, data were collected from five MAPs in five Canadian cities with each program constituting a case. In total, 53 program participants, 4 past participants and 50 program staff were interviewed. We used situational analysis to produce a series of “messy”, “ordered” and “social arenas” maps that provide insight into the social worlds of participants and the impact of MAPs.

**Results:**

Prior to entering a MAP, participants were often in a revolving world of cycling through multiple arenas (health, justice, housing and shelters) where abstinence from alcohol is often required in order to receive assistance. Residents described living in a street-based survival world characterized by criminalization, unmet health needs, stigma and unsafe spaces for drinking and a world punctuated by multiple losses and disconnections. MAPs disrupt these patterns by providing a harm reduction world in which obtaining accommodation and supports are not contingent on sobriety. MAPs represent a new arena that focuses on reducing harms through provision of safer spaces and supply of alcohol, with opportunities for reconnection with family and friends and for Indigenous participants, Indigenous traditions and cultures. Thus, MAPs are safer spaces but also potentially spaces for healing.

**Conclusions:**

In a landscape of limited alcohol harm reduction options, MAPs create a new arena for people experiencing severe alcohol dependence and homelessness. While MAPs reduce precarity for participants, programs themselves remain precarious due to ongoing challenges related to lack of understanding of alcohol harm reduction and insecure program funding.

## Introduction

Alcohol dependence has both short- and long-term health and social consequences. These include increased acute harms due to injuries and poisoning caused in part by periods of heavy use, chronic harms such as liver disease, cancer, strokes and gastrointestinal illness associated with long term consumption, and social harms related to housing, finances, relationships, law and the workplace [1–3]. Among homeless male populations, the prevalence of alcohol dependence has been estimated to be 37.9% compared with 3 or 4% in the general population [4, 5]. People experiencing chronic homelessness and severe alcohol dependence often experience poor mental and physical health (e.g. increased risk of suicide, depression, seizures and withdrawal symptoms, chronic harms related to liver function), premature mortality, violence (assault, theft, exploitation), have difficulty accessing and sustaining housing, and have limited access to health care resources and programs [6–9]. Heavy episodic alcohol use or binge drinking and consumption of non-beverage alcohol (NBA) further contribute to harms among homeless populations [10, 11]. Use of less expensive and more readily available NBA such as rubbing alcohol or mouthwash increases health risks due to additives in these products and the stigma often associated with drinking NBA [12]. Crabtree et al [13] describe “illicit drinking” as the consumption of NBA and/or drinking that is stigmatized and criminalized.

The situation of illicit drinking and related harms highlights the precarious nature of those living with severe alcohol use and homelessness and the extreme social marginalization experienced as a result. This combination of severe alcohol use and homelessness can be understood as being a situation of “precarious living”. “Precarity” as defined by Butler [14] is “the politically induced condition in which certain populations suffer from failing social and economic networks . . . becoming differentially exposed to injury, violence, and death” (p. 25). Rather than blaming or moralizing, “precarity” exposes the broader political and economic forces that create fragmented systems of care as well as “a path to understanding how those who are thrown into precarious circumstances find ways to live otherwise” [15]. Structurally violent forces include colonization for Indigenous people, capitalism that contributes to poverty and homelessness, and policies of exclusion and displacement on the basis of sex, gender and ethnicity. These violent processes are implicated in the production of severe deprivation, anxiety, stress, abuse, trauma and pain with substance use as a response to and way of managing life [16, 17].

Those experiencing severe alcohol dependence, poverty and homelessness have identified that abstinence is often not a realistic goal and that goals related to harm reduction are preferable and more achievable [13, 18–21]. Rhodes’s risk environment framework provides a rationale for harm reduction, shifting the focus and blame away from individuals and their behaviours, to the contexts of precarity produced by social, physical, economic and policy environments and levels of influence (micro and macro) that produce vulnerability and inequitable distribution of harms [22, 23]. Understanding the role of social and structural factors that produce drug-related harms is useful as a means of creating “enabling environments” for harm reduction and enhancing implementation of harm reduction programs that mediate and mitigate the risks for people who use substances [23]. As Rhodes observes shifting from individuals to environments as the focus of analysis brings into view a broader set of factors that influence drug-related harms and opportunities for addressing harms.

Some Housing First programs provide safer drinking education and accommodation while tolerating continued personal use of alcohol; providing an alternative to street-based drinking and harms [24–26]. Managed alcohol programs (MAPs) go one step further by providing and managing alcohol [21]. These programs aim to improve health and social outcomes by providing shelter or housing alongside regulated access to less hazardous forms of alcohol combined with social and cultural programming. MAPs have begun to thrive in Canada with more than 20 programs established (see www.cmaps.ca for an overview of MAPs in Canada). Research to date has found that MAPs have the potential to reduce consumption of NBA, stabilize risky patterns of drinking, reduce alcohol related harms, provide a sense of increased safety and security, and reduce contacts with police and emergency health services [12, 27–30]. Specifically, MAPs have been identified as safer spaces than hospitals, jails and treatment, and as spaces for recovery [31]. Beyond preliminary qualitative research suggesting that MAPs function as “safer environment” interventions, little is known about the impacts of MAPs for participants.

### Research purpose and questions

This analysis is situated within a larger program of research, the Canadian Managed Alcohol Programs Study (CMAPs) evaluating the effectiveness, implementation and impacts of MAPs in multiple Canadian cities. The purpose of CMAPS is to rigorously evaluate MAPs in Canada and generate insights into the impacts and implementation of MAPs in multiple settings. The purpose of this paper is to present findings related to the impacts of MAPs through an understanding of the social worlds of the participants (e.g. MAP residents and staff) and the social and structural shifts that occur pre MAP to post MAP.

### Research design and methods

We used a multiple case study research design and employed situational analysis (SA) as our approach to data analysis.

#### Case study methodology

Case study methodology is appropriate for understanding phenomena within a particular context that allows for an understanding of casual linkages through replication and pattern matching. In case study methodology, the triangulation of multiple sources of data, data collection at multiple settings and from different participants contributes to rigour and trustworthiness of the findings [32]. Stake [33] recommends use of at least three cases in a multiple case study design. In this study, there were five cases with each MAP constituting a case. We employed situational analysis as our approach to analysis for each of the five cases.

#### Sample and data collection

Data were collected between September 2013 and February 2015 through individual semi-structured and in-depth qualitative interviews lasting from 45 to 90 minutes with program participants and staff in five MAPs in five Canadian cities. Interviews were conducted by trained researchers and research assistants, audio recorded and transcribed. Ethical approval for this study was obtained from the University of Victoria (Protocol 13-002), as well as academic institutions of co-researchers and the various MAPs. Interviews were completed with 53 current program participants, 4 past program participants and 50 program staff. Current and past program participants were between the ages of 25-74, of which 43 identified as male and 13 as female. Twenty-three program participants identified as White, 23 identified as having First Nations, Métis or Inuit ancestry, four identified as other visible minorities and seven declined to answer. On average, staff reported approximately 2 years working experience in MAPs. The majority of staff had completed or partially completed college diplomas (34%), Bachelor’s degrees (24%) or graduate degrees (22%).

#### Analysis

Situational Analysis (SA) is a methodological expansion of grounded theory (GT) developed by Adele Clarke (2005) [34]. According to Clarke, SA helps to “(re)ground grounded theory after the post-modern turn” (p.52) while integrating poststructuralist perspectives for understanding human action, agency and structural contexts within complex situations. Thus, SA expands on the outcome of theorizing a basic social process in GT and instead focuses broadly on the “situation” as a non-reducible system of human and non-human elements, including historical, temporal, geographical, social and political aspects of the situation, its structural conditions and discourses. SA implements lenses of power, vulnerability and oppression in order to consider how representations of individuals or collective actors may be constrained by discursive construction. Critical to the postmodern roots of SA is a shift from homogeneity to complexity, difference and heterogeneity.

The shared commitments of individual and collective human and non-human elements reflected in data sources are visually mapped in SA in order to bring the researcher to consider complexity, emergence and variation within the situation at hand. In situational analysis, three types of maps are produced [35]: *situational maps* that lay out the major human, non-human and discursive elements in the situation of inquiry (messy and ordered maps); *social world maps* that lay out the collective actors, key non-human elements and the “arenas of commitment”; and *positional maps* that lay out the major positions taken vis-à-vis particular axes of difference, concern and controversy around issues in the situation of inquiry. To begin SA analysis, we employed the constant comparative method of GT for line by line coding and categorization of the interview data [23]. Through analysis and reading of the data, researchers can identify human and non-human, sociopolitical, temporal/historical and discursive elements through what is both said and not said by participants and identifying the “taken for granted”. Initially, situational maps were developed for each case as an analytic strategy to open up the data, followed by the development of social worlds maps.

In this paper, we present social world maps as we wanted to understand the impact of MAP on the social worlds of participants pre and post MAP. According to Clarke (2005) the utility of these maps is gaining a collective sense of the situation, particularly in regards to how people organize themselves and participate in (re)producing discourses among broader structural situations. Social worlds are meso-level analyses that allow the researcher to view collective social action, in our case the impacts of MAP from the perspective of participants pre and post MAP. Multiple perspectives and commitments, including their overlaps and tensions, are signified through the porous boundaries of multiple sites of action. According to Clarke, social world maps can represent the “big news” of the situation at hand. Understanding and mapping discourses relevant to the production and maintenance of a given situation is essential to SA with “social worlds” representing “universes of discourse” [35]. Using SA allowed us to “map” the shifts and impacts pre-MAP to post-MAP, through participants’ presence and participation in both organized collectives (arenas) and socially constructed discourses (worlds).

#### Cross case analysis

Following the development of situational and social world maps for each case, we used techniques of cross case comparison to develop a social world and arena maps for the overall situation. Aided by NVivo, cross case analysis was completed using data generating techniques proposed by Miles and Huberman [36] which support systematic analysis of commonalities and differences within and across cases. Through the use of case study methodology and SA, we were able to identify both similarities and differences within and across cases to gain insight into pre-MAP and post-MAP arenas from the perspectives of those participating in MAPs.

## Findings

### Pre-MAP arena: displacement and survival in abstinence-based worlds

**Fig. 1:**
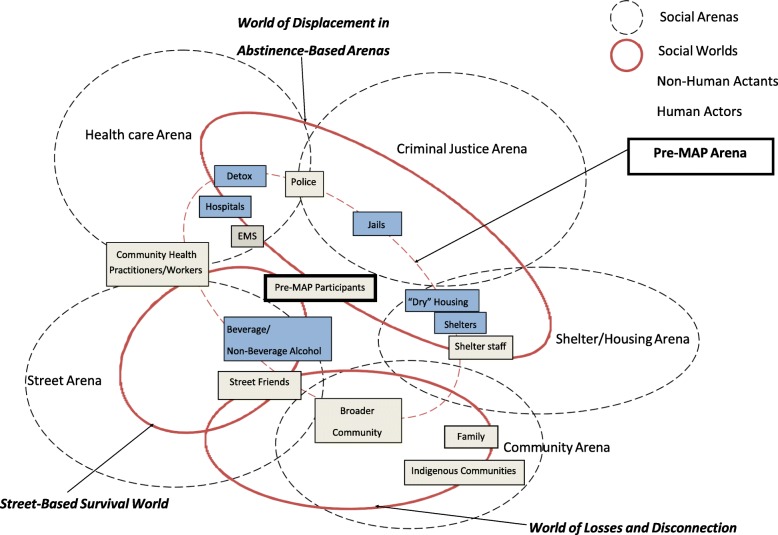
Pre-MAP social arenas. Prior to entering MAP participants cycled through multiple social arenas (black dotted circles) including health care, justice, shelter and housing, community, and street arenas. The experiences of MAP participants within and between arenas are characterized by social worlds (continuous red circles) shaped by “universes of discourse”, including a World of Displacement in Abstinence-based Arenas, World of Losses and Disconnection and Street-Based Survival World. Individual and collective human actors (grey squares) and non-human actants (blue squares) significant to the situation are positioned according to their roles in the production of social worlds

Prior to coming to a MAP, residents described their past experiences of alcohol dependence and homelessness as living in a daily cycle of displacement moving from streets to shelters, jails, hospitals and at times treatment. The pre-MAP arena represents the intersection of multiple arenas including justice (policing and jails), health care (hospitals, detox, treatment and emergency departments), housing and shelters and is comprised of several *social worlds*: (1) a world of displacement in abstinence-based arenas; (2) a street-based survival world and (3) a world of losses and disconnection. Below, we present the social worlds in the Pre-MAP Arena as outlined in Fig. 1. Throughout the findings, direct quotes are provided from MAP resident and staff participants using assigned pseudonyms.

1.1. World of displacement in abstinence-based arenas MAP residents often characterized their lives before entering a MAP according to frequent and transient contacts with multiple systems or arenas including justice (police and jails), health care (emergency, acute care and detox), housing and shelters with multiple discursive practices of displacement.

“Ah when I used to stay on the streets and there, sometimes I didn’t even drink the whole day and just tired and needed a place to rest. ‘Cause you know I got the ambulance picking me up or the cops waking me up, taking me to jail, you know hospital or detox” —Marlene, 47, MAP Resident

Residents described multiple encounters with police and emergency personnel, who often diverted them from the streets to other locations such as shelters, hospitals, detox or jail depending on the particular situation. Displacement and revolving– from streets and shelters into and through hospitals, treatment and jails—for some was a daily experience.

Residents reported encounters justice arenas and histories of charges or fines related to “public intoxication” and “pan handling”.

“When you’re out on the street, or taking care of yourself, you just make that move to go back to the [liquor store] an hour later and then by two or three o’clock in the afternoon, you’d be on the park bench half in the bag and sound asleep, and wake up in the drunk tank.” —Frank, 54, MAP Resident

Additionally, laws or bylaws related to street drinking involved police interactions with the outcome of ending up in jail, as in the example above. Violence or aggression in housing and/or shelters as well as shelter or housing policies that restricted alcohol and other substance use resulted in bans from services. These discursive practices of displacement contributed to and increased criminalization as opposed to providing assistance and addressing structural inequities, albeit in some cases police took individuals to hospitals and/or shelters as alternatives to jail.

Prior to MAP, residents often cycled through emergency and acute care environments as a result of ongoing health issues, risky drinking patterns (e.g. withdrawal seizures, injuries), lack of primary care and homelessness. One participant described:

“I was on the streets when I came here [MAP]. I came from a psychiatric ward, but I was on the streets before that and I was really messed up. Paranoia, taking pills. I’d get my prescription and I’d take it all at once. And, pass out and end up in the psychiatric ward again. And, or the emergency ward. Every week I was in the emergency ward, for seizures.” —Owen, 38, MAP resident

Although this participant and others were frequently interacting with and cycling through health systems, rarely were their health care needs met. The challenges of homelessness in the context of premature ageing with a physical disability, lack of primary care and access to medications, complicated the struggles of participants living within constantly shifting and insecure conditions.

MAP residents described multiple and frequent contacts with detox facilities or treatment centres as part of cycling through a primarily abstinence-based arena. Residents cited histories of multiple and repeated attempts to complete detox and/or rehabilitation.

“I’ve been to AA, yeah. I went to […] a dry house here, and I was sober for five months. And…after doing the program, actually twice over, and everything, like I stayed in there two times for the length of the program. I just got- there was just internal conflict again and I end up just saying, ‘Okay, I’ve had enough, I’m going out and leaving’ and relapsed, drank the same day. Had a two month bender there and ended up in the hospital.” —Albert, 59, MAP resident

Residents with past experience in abstinence-based peer-support programs, such as Alcoholics Anonymous (AA), variably spoke of the role of these programs in their lives. In the quote below, a resident describes their past experiences of judgement and guilt that deterred their further engagement in AA:

“Well I’ve been involved with AA for a long time […] but you screw up once, you start all over again. You don’t get that ‘You know what you were sober for seven months, you slipped, but we’ll start again” it’s kind of like “You screwed that up big time, and you’ve lost everything you’ve worked for. All that time you had under your belt is gone’ […]I think in AA, when- how they harp like that, about time lost and…it makes you that embarrassed and ashamed, you don’t want to go back. Guilty. Ashamed, embarrassed.”—Quinton, 51, MAP resident

In this example and others, residents highlighted that in abstinence-based programs they experience feelings of judgement, shame and blame. This had the opposite and unintended effect of sending people back to the streets and drinking when they were unable to sustain sobriety.

In the pre-MAP arena, MAP residents lived in a world of displacement, cycling through multiple systems including health care, justice, housing and shelters characterized by abstinence, criminalization and ongoing instability, with systems failing to address health issues, and at times contributing to negative feelings such as shame and guilt and the precarious nature of their lives. Although they had frequent contact with systems, in some cases multiple systems, their health, safety and housing needs were largely unmet.

### Street-based survival world

Residents described their coping in a world of displacement as living in the stress of survival—a street-based survival world.

“Yes. Stress of survival. I mean, when you live on the streets, you’re always thinking forty-seven scenarios ahead because you’ve got to figure out what you’re going to do with your next so many hours; how you’re going to maintain what you want to do or where you’re going to live. You know, is there going to be a bed available if you miss the night before? You know, all those factors go in and, oh, is it going to rain today? I got to make sure I’m sober enough that I can get into the shelter because it’s going to be raining and I’m going to be out in the rain all day so I’m going to need to go in and get a good night’s sleep and dry off.” —Geoff, 46, MAP Resident

As this participant points out survival requires thinking ahead and one has to plan for periods of drinking and sobriety in order to manage getting through the day (survival practices). A key aspect of survival living is ensuring one has enough alcohol to sustain themselves. Some participants described their own attempts at employing harm reduction practices in the absence alcohol harm reduction services that would otherwise provide safer spaces or sources of alcohol. For example, participants described rationing alcohol throughout the day, storing alcohol in secure places to be found later, or “pan handling” to gather funds for beverage alcohol. However, as the resident describes below, maintaining these harm reduction practices proved difficult on the street with insecure funds.

“So, pan handling on the street and waiting. What’s happening for you when you’re waiting? (Interviewer)

I’m shaking. I’m throwing up. I’m hallucinating, and it’s- I find it tough because you’re getting frustrated because you can’t get that next drink. And, sometimes when the wine rack closes, they got no choice but to find a twenty-four hour store, either buy the rubbing alcohol or Listerine. You know, that’s all we get for a drink. And I don’t want to be drinking that…that…stuff anymore.”—Herald, 43, MAP Resident

Residents described drinking larger amounts of alcohol before entering a MAP and binge drinking with acute physical harms, such as falls and passing out in unsafe spaces. When alcohol was not available and financial means for the purchase of beverage alcohol were limited, participants often lived in risk of alcohol withdrawal (and the potential for seizures) and the need to find ways to prevent withdrawal through obtaining and consuming illicit alcohol such as rubbing alcohol, mouthwash and hand sanitizer. Participants, like the one above, reported going to extreme lengths such as stealing to prevent these physical harms, often resulting in further harms of criminalization (e.g. arrest, incarceration).

An often-cited example of social connection in the pre-MAP world was drinking in the park or other public spaces with friends. While participants sometimes discussed the realities of living in the pre-MAP arena such as being in close quarters with others, the realities of theft and violence, many participants spoke of their street friends as a primary source of connection, support, activity and protection. As one participant described, building relationships on the street enhanced security and safety in shelter settings:

“You kind of hang out with certain people. Or …you know, even if it’s a dorm setting, they’ll be sleeping in the next bed to you or something, you watch each other.” —Michael, 36, MAP Resident

Some residents described their friendships on the streets as akin to a ‘brotherhood’ or ‘street family’. This family provided protection but also sharing of alcohol and a source of support in a highly stigmatized abstinence-based arena.

Staff reflected on the stigmatizing ways that MAP participants are described in the community such as being the “hardest to serve”, when in fact they are essentially slipping through the cracks of a system in which there are little to no spaces to live and maintain their health while also drinking alcohol.

“The population that comes to, to, say, managed alcohol, they tend to be outliers, they, they tend to not conform to current programming or more, mainstream programs. So they’re viewed as being a disruptive influence rather than, just having different needs” —Tanya, 38, MAP Staff

In this street-based survival world, it is difficult or impossible to secure stable housing, save money, have regular access to alcohol to prevent withdrawal, ensure safety of oneself and one’s belongings, or maintain connections with families of origin and communities. Thus, residents live in a precarious world that is focused on survival needs. The precarity of street survival is somewhat mitigated by individual harm reduction practices and relationships with street friends and family. The primary alternatives to the street-based survival world are abstinence-based programs which, as described earlier, are often associated with feelings of shame and blame and do not mitigate risk environments of those with severe alcohol dependence and homelessness.

### World of losses and disconnections

For MAP residents, the pre-MAP arena was marked by multiple losses and intense exclusion and disconnection from family and friends. In the following quote, one MAP resident contemplates trying to reconcile with their family and the past:

“My sister called downtown…I don’t know, a year ago, she’s looking for me too […] I could go see her. But I’m embarrassed about that too […] she gave me all kinds of money to rent a place and for- I never paid her a dime back. Just out drinking. But I don’t think it’s the money, she just wanted to know if I was alive or not” —Donna, 48, MAP Resident

As above, participants identified their street families as strong sources of connection, although these relationships were also associated with significant loss and harmful patterns of drinking:

“I’ve lost, like I don’t know how many people, this- of my street family- this year. And each time I lose one of them, and I think that, you know, it’s going to be me next […] where what we do is we go out and get drunk. And I don’t want to handle it that- like, that way anymore, you know what I mean?” —Tony, 55, MAP Resident

In this quote, the resident reflects on the death of their street family as illustrative of the significant harms of homelessness and street-based alcohol use and further connected these experiences to their own relationship with alcohol. Participants often described significant and traumatic life events, such as traumatic physical injuries, abuse or death of family or friends on the street that triggered their homelessness, alcohol use or otherwise exacerbated situations of precarity. Loss and grief were a significant part of participants’ worlds.

Indigenous participants described the loss of Indigenous traditions and practices as well as the loss of connections to their community and families. One participant highlighted their disconnection from Indigenous traditions and culture:

“I believe in my culture and my traditions and plus the creator and I lost that, you know. I lost that part there where we would you know smudge in the morning and you know and say thank you to our creator and then somehow I just quit doing that. Quit praising, quit praising our creator, I used to ah be able to you know join the celebration; you know there’s powwows and all that. I don’t even do that anymore you know.” —Daniel, 41, MAP resident

The Indigenous MAP participant above describes the loss and disconnection from Indigenous culture and traditions as a result of homelessness. Such experiences must be placed into the historical and ongoing context of colonization in Canada. Past and current contexts of colonization have resulted in the loss of lands and resources of Indigenous peoples, the creation of residential schools, Indian hospitals, 60’s scoop and the current foster care system with ongoing processes of colonization through incarceration and racism [37]. Some residents described experiences of displacement from family and ancestral communities that impeded connections to tradition and culture. Residents from rural ancestral communities faced geographical barriers to connecting with their cultural communities, as they are often living in larger urban centres. These experiences are consistent with several of the dimensions of Indigenous homelessness in Canada [34] including “cultural disintegration and loss” homelessness, “relocation and mobility” homelessness and “historic displacement” homelessness.

Cycling through health, justice and housing systems positions individuals to live in a precarious world of street-based survival with ongoing disconnections and multiple losses characterized by criminalization, stigma, abstinence, unmet health care needs, unsafe drinking patterns and spaces with ongoing precarity. The primary supports are individual harm reduction practices and street friends/family, thus highlighting a significant gap in services for a population impacted by structural violence with vulnerability to harms of alcohol use exacerbated by homelessness. Indigenous residents specifically expressed their feelings about the loss of Indigenous culture and traditions highlighting the socio-political and historical factors that have shaped Indigenous homelessness.

## MAP arena: “There is a Place”

**Fig. 2:**
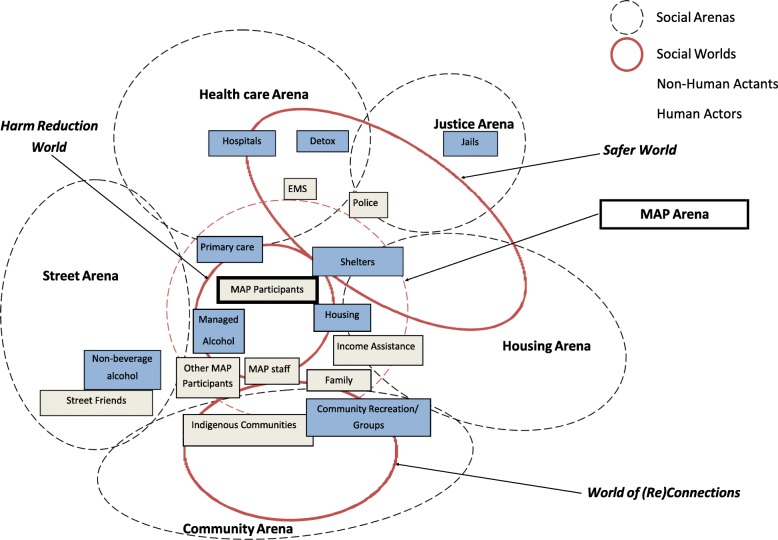
MAP social arenas. The introduction of the MAP arena shifts the cyclical movement of MAP participants within multiple social arenas (black dotted circles) towards primarily the MAP arena. Experiences of MAP participants within these arenas also shift from Pre-MAP social worlds toward new social worlds (continuous red circles) shaped by “universes of discourse”, including a Harm Reduction World, Safer World, and World of (Re)Connections. Individual and collective human actors (grey squares) and non-human actants (blue squares) significant to the situation are positioned according to their roles in the production of social worlds

The creation of a MAP represents a new arena that did not exist previously for participants who were constantly being displaced and cycling through largely abstinence-based arenas. MAP participants were often referred or admitted to a MAP from a situation of homelessness such as living outside, temporary shelter or housing instability (e.g. Couchsurfing). As depicted in Fig. 2, the MAP arena consists of (1) a harm reduction world, (2) a safer world and (3) a world of re-connection. Central to the MAP arena is a harm reduction world which provides an alternative to largely abstinence-based worlds available pre-MAP as well as a safer, less precarious world with more supports and enhanced social connections.

### Harm reduction world

Transitioning into a MAP is heavily contingent on safe, non-stigmatizing and welcoming relationships and spaces. In every MAP, what residents valued most was the respect and care they received from MAP staff. Residents regularly compared their interactions with MAP staff to their past experiences in other programs:

“Just the way- they don’t belittle us. They, you know, like- I feel human, whereas if you’re out somewhere and you meet up with other people, they say ‘He’s an alcoholic, he’s got a problem with alcohol, whatever’, they start making you feel belittled. But here, they just treat you as a human being, which I like. So I feel comfortable.” —Leon, 53, MAP Resident

According to MAP staff, the relational component is integral to working in a MAP. They related the importance of countering previous experiences of stigma, loss of self-determination and dignity with respect and caring. Residents echoed the importance of respect for choice and autonomy:

“Because the worst thing you can do to a drunk, don’t push him against the wall, because he’ll come out fighting. Like don’t corner him. Give him a wide berth, because he will come to you when he’s ready to come to you, don’t try and jam- go down his throat and “Oh yes, you’re going to get healed whether you like it or not” ah ah ah, that doesn’t fly with us. No. Because now you’re taking away our right to be who we are. This way, here, what they’re doing here, is they’re giving your right back. They’re giving it to you, they’re saying “It’s yours, it’s yours. Take it. It belongs to you. It’s you.” —Ronald, 37, MAP Resident

This resident describes being able to be who they are in the MAP arena signifying a hallmark of harm reduction practices which is the provision of non-judgmental care and unconditional acceptance regardless of substance use [38]

In the MAP arena, residents were not required to be abstinent in order to access health services and supports. MAPs provide a space for drinking and provision of safer sources of alcohol.

“So that’s one of the good things I like about this program, is they- it’s a managed alcohol program, so they kind of manage the way things are going with respect to your drinking, your eating, your health habits, your cleanliness. So you put all of them in- all of them in line and it gives you a little bit of hope in relation to the future.” —Frank, 54, MAP Resident

This participant also highlights essential determinants of health such as nutrition, a place to sleep and resources for hygiene closely linked to managing drinking as part of reducing harms and contributing to hope for the future. This is in direct contrast to the pre-MAP arena where their needs were largely unmet with little hope for the future. However, access to social and health supports was not consistent across MAPs. In some programs, a lack of around the clock nursing and personal care supports increased resource demands on non-clinical MAP staff. In these programs, staff and participants described continuing encounters with abstinence-based services including emergency and hospital services, where social harms of stigma and discrimination related to alcohol dependence are ongoing.

From the perspective of staff, working in the harm reduction world was challenging because they often had to navigate outside relationships in the abstinence-based arena when residents needed health or other services outside of the MAP.

“So for us, in terms of contact with the medical community, that’s where we’ve been pushing. It’s like, it’s not so much that our guys need to change. We felt that the medical community needed to, kind of, broaden its horizons a little bit. In terms of…. fundamental harm reduction sense, working with people where they are.” —Justin, 28, MAP Staff

Alcohol harm reduction was not necessarily widely accepted or understood outside of the MAP arena by those in emergency departments, housing, police and even by other harm reduction programs for illicit drug use. Staff have to navigate these challenges when clients go to the hospital as well navigate with police if clients are found “pan handling” or drinking in the community (brought back to the program). Thus, an important aspect of implementation is alcohol harm reduction education for other organizations as the goals and impacts of MAPs are not always well understood.

### A safer world

The harm reduction world intertwines with other aspects of MAPs to create a less precarious, safer world, as is illustrated in the following excerpt from staff:

“I’d also consider we’re talking about those sort of three prongs, the alcohol intervention, their mental health and well-being intervention, their housing and community intervention. And make sure you’re really targeting all three of those at the same time. And that one doesn’t necessarily weigh heavier than the others. So, your right to your housing should not be contingent on your MAP program, your MAP program is not contingent on you taking part in, you know, mental health counseling programs. Things like that, that all three are equal and that you need all three to be working together to have complete wrap-around care for a participant.” —Devin, 43, MAP staff

A primary shift associated with transition into a MAP is that residents no longer need to chase alcohol to survive and have a safer place in which to consume alcohol.

“ ‘I’m tired of chasing the beer’. It…it…it’s…it’s a mental thing [...] you can sit there and watch a TV show and not go bang, “Okay, where’s my next one?” … It takes away a lot of the stress. It takes away the anxiety, it takes away….all the street factors.”—Bruce, 32. MAP resident

The resident below emphasizes the importance of the MAP as a safer place that reduces harms:

“I can sit here and drink a cup of wine. It reduces the harm by making it that you don’t really have to go out. And if you do have to go out, you might go to the beer store and get a couple of beer. Not to the hospital and rip off jars of hand sanitizer. Go to Shoplifters Drug Mart and steal Listerine, which, it eliminates. I think that’s reducing harm. Plus, you’re out on the street drinking, and chances of getting into trouble and violence and violent trouble, it’s reduced because you’re not out there drinking.” —Moses, 63, MAP resident

Residents generally described feeling safer or more secure in the MAP as described below:

“When I was downtown, I would be so drunk I would get in trouble with the cops, with other people, fights and all that. But here, it’s very…you don’t have to prove yourself for anything here. Downtown you always have to be looking behind your shoulder and…and…not here, it’s very safe here. And it’s like a home environment […] because, home is safe; home is where you want to be and home is always there. Where the place you call home is where you live, and I live here. And I feel that I live here.” —Jonathon, 45 MAP Resident

While entrance into a MAP is associated with increased security and access to safer spaces and sources of beverage alcohol, there are ongoing issues related to implementation we will discuss elsewhere including transitioning from the street, violence and aggression associated with drinking, alcohol management policies and practices, and historical and ongoing conflicts between residents. Here we highlight structural conditions impacting the maintenance and sustainability of the MAP arena.

MAPs differ in their housing models including residential permanent, transitional and shelter models [21]. For example, participant descriptions of shelter-based MAPs ranged from programs as “home” to just a place to sleep, albeit a place that is relatively safer than the street. Some participants in shelter-based programs also illustrated ongoing experiences of insecurity being in close proximity to the street and in a shelter:

“Overall, yes [I feel safe]. But I’ve always got my backup…I think it’s the atmosphere. It’s a homeless shelter. I’m in a room with alcoholics, every- I don’t know what they’re up to, what they’re using, so you’re always kind of- yeah, you can’t totally sit and just say ‘Phewf, whatever’ you got to kind of…not be on your guard, but just kind of sense- your senses are a bit higher.” —Quinton, 51, MAP resident

Participants in transitional programs also experienced anxiety regarding their tenure and security in housing. Program staff repeatedly highlighted the challenges of finding permanent housing for participants transitioning out of programs, as well as obtaining stable ongoing sources of funding for the MAP. Staff described this process as a constant battle to chase funding in order to continue operations or evolve programs in order to suit available funding sources. These issues are highlighted here to illustrate the ongoing economic and social conditions which continue to contribute to ongoing precarity for participants.

### World of (Re)connections

MAPs provide an opportunity to maintain, build and connect social supports that are central to healing and recovery. Transitioning into a MAP alongside a street family who looks out for each other’s best interests was reported as helpful by several residents.

“We brought each other in here […] so all four of us that would normally be together out there are all in here. And we’re all seeing changes in each other. We’re seeing each other for what we really are, not from what we saw before. Before it was just a drunken haze.” —Ronald 37. MAP resident

As this participant describes, a street family remains an important source of support and maintaining social capital and relationships with street family facilitated their transitions into the program. However, sometimes street relationships could not be maintained, requiring the development of new social connections as participants moved into MAPs. A particular struggle for residents was maintaining or distancing relationships with friends outside of MAPs who may still be drinking erratically or drinking non-beverage.

“Well, well first of all I’m not drinking hairspray, Listerine, mouth wash, hand sanitizer, cologne, body… body spray or anything that contains alcohol. I haven’t touched that stuff […]. But that ah I have to limit my visits with my friends now cause they’re still, still doing ah the ah other stuff, which I’m trying to stay away from but I still talk to them and they see the improvement on me right… Ah they congratulate me after a while. Then once I sit down with them, I’ll have a smoke or a cup of coffee while they’re drinking, was they start bad mouthing me, there a I say ‘Ok it’s time for me to go, I’ll see you guys later.’” —Marlene, 51 MAP resident

A sense of connection to others in the program and feeling of community were described by residents and staff as important to MAP operation and important to feelings of connection and social support. At some sites, participants shared meal planning and chores. Recreational activities provide a medium for staff to engage with residents and were often cited as key coping mechanisms. At some sites, residents described being in the MAP as similar to living a cooperative in which everyone participates, or feeling that the program was like “family” as illustrated by the following description from staff:

“…there’s camaraderie […] you spend a lot of time with people, and they drink, and all of a sudden they’re playing cards, they’re watching TV together, they sleep in the same places. They, you know, day in and day out, and all of a sudden you see friendships developing and then all of a sudden they’re all saying this is more than just friendships. They classify themselves as family.” —Stephen, 54. MAP staff

Developing a family culture for participants who may have little support outside the program was identified as important by residents and staff:

“It’s like [clears throat] that there was a part of me missing when I was out on the streets. I pan handled, dig in the garbage, I’d steal food - just to survive. And like I didn’t have anybody, like… like when I’m here like I have everybody and I try to make everybody happy. But when I was on the streets, it was just me, I didn’t have anybody to feed, didn’t have anybody to make happy, all I talk about is just myself. When I’m here I think about everybody. Make sure they’re ok, make sure they’re fed, make sure they take their medication.” —Marlene, 51, MAP resident

Beyond developing connections within the program, the security and safety provided by MAPs facilitated a sense of home and permanency. Through provision of accommodation, as well as stabilized patterns of alcohol use, reconnection with self and others started to become a possibility.

“And now I’m unloading my garbage and um I guess you could say I’m ah recycling myself. You know get rid of the garbage and start putting good things in me you know, like getting to know my children…”—Louise, 41, MAP resident

These possibilities for reconnection included reconnection with self, family, community, police and culture. Reconnecting with family was a milestone for some participants and a future goal for others. While not consistent across MAP sites, staff generally noted that neighbours and community members were supportive of MAPs and interacted with programs from time to time, such as by providing donations. Across some sites, MAP staff reported decreased contact and improved interactions between participants and police officers, rebuilding more positive connections with officers who are supportive of MAPs in their communities. Indigenous MAP participants identified the importance of reconnecting with their community as part of the healing.

“…then I try to get back involved in the native community. That’s what they’re pushing for. To smudge and smoke sweet grass. […] We’re working on it […] but to be with my own people would be…I think that would help a lot. Take some of the pain away.” —Malcolm, 31 MAP resident

Although none of the MAPs in this study were Indigenous-led, there were specific examples of providing opportunities for Indigenous cultural supports such as working with an elder or participating in events or ceremonies to rebuild connections with their ancestral communities and cultural identity.

## Strengths and limitations

One of the main strengths of this research is that it triangulates multiple sources of data from multiple perspectives and across settings to enhance the rigour of the analyses. However, like all qualitative research, it is not possible nor desirable to generalize findings. All of the programs in this study were developed largely by service providers with varying degrees of input from potential MAP participants. Also, each site was unique in terms of the conditions and context in which the MAP was being implemented, and so, in creating cross case situational maps, there is the potential for loss of unique programmatic differences in multiple settings in the pursuit of generating a common understanding. Further, programs are constantly evolving in response to clients’ needs, program leadership and funders.

## Discussion

People experiencing chronic homelessness and severe alcohol dependence are differentially exposed to injury, violence and death with difficulty accessing and sustaining housing and limited access to health care resources and programs [12, 13]. In describing the arena of displacement and survival, our analysis provides insight into these precarious living circumstances by detailing overlapping patterns of cycling through housing, health, and justice systems and street-based survival. Residents experienced criminalization, stigmatization, unmet healthcare needs, unsafe drinking environments and patterns of drinking with increased feelings of guilt and shame when accessing abstinence-based services. Their supports were largely survival strategies including individual harm reduction practices as well as protection and support through connection with street friends and family. MAP interventions provide a new arena for those surviving in high-risk environments and precarious circumstances [15] through creation of a new arena composed of harm reduction and safer worlds, providing opportunities for reconnection to family, community and culture. Impacts for residents include feeling accepted, respected, safer and hopeful as well as increasing access to services, increased stability, safer space for drinking and safer drinking patterns, and reconnection with self, family, and social and cultural community. In other research, the creation of the MAP arena has been found to be cost effective as it reduces this cycling through multiple systems with higher costs as well as being more effective in connecting people to services [30].

MAPs are introduced as an alcohol harm reduction intervention in a continuum of largely abstinence-based arenas and an alternative to the street-based survival world, disrupting the constant cycle of displacement through multiple arenas where individuals needs are largely unmet. MAPs provide an alcohol harm reduction intervention to meet the ongoing needs of people experiencing chronic homelessness and severe alcohol use disorders. Harm reduction programs seek to reduce the harms of substance use without necessarily eliminating use while also emphasizing trust, dignity and respect [39–41]. Like other harm reduction interventions such as supervised consumption, MAPs provide a safer space for use as well as the provision of a safer source of substances similar to heroin prescription programs [42]. However, MAPs are on the margins of a harm reduction field dominated by illicit substance use and often receiving less attention than harm reduction services for illicit drug use. As well, alcohol harm reduction interventions for this population are often unrecognized in the alcohol policy world as an important intervention to reduce harms for those living in the context of poverty and homelessness [43]. In lieu of harm reduction spaces and services pre-MAP, participants drew upon harm reduction practices including peer support as well as strategies for safer alcohol consumption. However, findings from this paper indicate challenges to maintaining these practices within abstinence-based arenas and precarious living situations.

Pre MAP, physical environments (e.g. homelessness, and sleeping and drinking outdoors), social environments (e.g. stigmatization and racism), economic environments (e.g. insecure and inadequate income), and policy environments (e.g. criminalization, fragmentation of care) interpolated a social world of survival, constant cycling through services and ongoing displacement and disconnection. These intersecting “risk environments” influence individual vulnerability to alcohol-related harms and precarious living circumstances that are socially and politically produced [22, 23]. Enabling environments are characterized by harm reduction interventions aimed at reducing the harms of alcohol and drug use and mitigating harms through micro and macro levels of influence to reduce vulnerability [44]. The MAP arena is introduced into a context of precarious living, creating a new arena that brings together housing/shelters, health care and harm reduction worlds thereby reducing the risks perpetuated by existing physical, social, economic and policy environments. MAPs reduce precarity within multiple “risk environments” [22, 23] and have been described as “enabling places” [31] or “safer environment interventions” [44]. In policy and practice, these findings highlight that while MAPs provide a safer, more stable world for clients, they often continue to operate within a precarious economic environment in which there is a lack of stable funding for programs [21]. In this paper, we have focused on the context in which MAPs operate and discuss program implementation elsewhere. This study contributes to the existing body of literature that highlights the importance of introducing harm reduction interventions to provide space and mitigate risk environments for people who use illicit drugs and alcohol [45, 46] and a space that supports possibilities for health and healing. Several authors have noted the importance of extending our thinking beyond risk environments to reduce drug-related harms to promote research and knowledge related to enabling environments that promote health and healing [45, 47].

MAPs exemplify the creation of a new space that does not require sobriety and abstinence in order to access housing. This is important for a group who is often the most likely to have difficulty finding housing, and among those least likely to find success in often highly successful Housing First programs [48, 49]. MAPs are aligned with some of the principles of Housing First, such as those that prioritize placement into permanent housing without requiring sobriety, yet harm reduction interventions such as MAPs are often seen as separate interventions lying outside of the Housing First arena [49]. Explicating the MAP arena highlights new insights for thinking about harm reduction spaces as intersecting and overlapping with other spaces and the need for understanding of harm reduction across multiple arenas as important to the implementation of harm reduction interventions as part of Housing First and as separate interventions [49].

Within MAPs, implementation requires specific attention to the consistent application of harm reduction principles that emphasize non-judgement and unconditional acceptance, development of trust and meaningful relationships as well as understanding the shifting, albeit continuing, relationship with alcohol. The impacts, from perspectives of MAP participants, are evidenced by participants’ experiences and feelings of acceptance, respect, choice, increased stability, safety, security and connection. These impacts are consistent with an earlier evaluation of a single MAP that facilitated improved feelings of safety as well as feelings of family and home. Opportunities for reconnection with other MAP residents, family, friends and community are consistent with the literature on therapeutic landscapes in which physical and social environments are conducive to health and healing [50, 51]. Thus, MAPs can be spaces that mitigate risks but also spaces that can promote health and healing. Importantly, attention is needed for the development and implementation of MAPs that is foregrounded by Indigenous approaches to health and healing.

## Conclusions

The MAP arena repositions MAP participants in relation to systems of care, alcohol, housing, friends, family and the community in ways that contribute to increased safety and security. MAP interventions mitigate but do not erase the precarious nature of peoples’ lives. MAPs have a precarious existence in relation to existing in a context in which alcohol harm reduction is relatively marginalized and poorly funded. Our analysis draws attention to both risk environments that produce vulnerability to alcohol-related harms and the importance of creating harm reduction spaces that mitigate risk but that are also healing environments. These spaces are characterized by nonjudgement and the opportunity for people to experience acceptance and safety with feelings of stability and security as well as access to resources and reconnection with themselves and others.

## Data Availability

The datasets generated and/or analyzed during the current study are not publicly available due the small sample size and associated threats to confidentiality, but are available from the corresponding author on reasonable request.
